# Coding and Non-Coding RNAs, as Male Fertility and Infertility Biomarkers 

**DOI:** 10.22074/IJFS.2021.134602

**Published:** 2021-06-22

**Authors:** Fereshteh Aliakbari, Nahal Eshghifar, Reza Mirfakhraie, Parisa Pourghorban, Faezeh Azizi

**Affiliations:** 1 Men’s Health and Reproductive Health Research Center, Shahid Beheshti University of Medical Sciences, Tehran, Iran; 2 Department of Cellular and Molecular Sciences, Faculty of Advanced Sciences and Technology, Tehran Medical Sciences, Islamic Azad University, Tehran, Iran; 3 Department of Medical Genetics, Faculty of Medicine, Shahid Beheshti University of Medical Sciences, Tehran, Iran; 4 Department of Biology, Faculty of Biological Sciences, Sabzevar Branch, Islamic Azad University, Sabzevar, Iran; 5 Non-Communicable Disease Control Department, Public Health Department, Ministry of Health and Medical Education, Tehran, Iran

**Keywords:** Male Infertility, Semen, Spermatogenesis

## Abstract

Semen analysis is usually the first step in the assessment of male fertility. Although analyzes provide valuable information about male fertility, success of cytoplasmic sperm injection using this method is not predictable. In the recent years, studies have shown that sperm quality assessment helps clinicians predict male fertility status based on the expression of biomarkers. To write this article, a comprehensive study was conducted on several RNA transcripts by searching related words on medical information databases by 2018. According to the literature, spermatogenesis based disorders in male infertility have a significant relationship with the expression level of some RNA molecules (like *DAZ* and *PRM1/PRM2* ratio) in semen and testicular tissue. Thus, they might be used as predictor biomarkers to evaluate success rate of testicular sperm extraction (TESE) procedure, but confirmation of this hypothesis requires more extensive research. By comparing the number of RNAs attributed to each fertility disorder in men, it is possible to trace the causes of disease or return fertility to some infertile patients by regulating the mentioned molecules. Further researches can provide a better understanding of the use of RNA expression profiles in the diagnosis and treatment of male infertility.

## Introduction

Sperm RNA contains several coding and non-coding transcripts that represent a picture of past events, such as spermatogenesis and sperm maturation. This, they provide new insights for male fertility and infertility research. On the other hand, a new scope in infertility study is participation of sperm RNA in the epigenetic transgenerational inheritance of the altered phenotypic traits in the progeny associated with paternal exposure (1). One of the main problems in infertile men is reduction of normal sperm cell quantity. Currently, despite the importance of sperm movement in the reproductive process, limited information is available about the molecular mechanisms related to sperm motility. Nowadays, new strategies for treating spermatogenesis of infertility, such as intracytoplasmic sperm injection (ICSI), reduce sperm disorders and sometimes easily recover it. Despite utilizing these methods can help resolve the infertility problem, the risk of transferring genetic problems to the next generation still exist. The main emphasis of molecular evaluation and analysis of RNA sperm is the important role of male factors in idiopathic infertility and difficult testicular biopsy procedure. These cases can also be useful as predictors of male infertility. It is estimated that about 35% of cases in infertile male are caused by genetic factors (2). More than 30 years ago, presence of RNA in sperm had been the subject of argument. The concern in this issue has recently expanded, due to the development of modern molecular technologies and the need for designing non-invasive methods for studying and assessing testicular function. If it is possible to obtain useful information about molecular events of sperm, a semen analysis will be a non-invasive approach compared to testicular biopsy. Today, with remarkable advances in molecular medicine, study of the sperm RNA content is growing using techniques that simultaneously examine expression of large number of genes, such as RNAseq and microarray. In recent years, study of effective genes in the male infertility process has been considered, due to their important role in therapeutic planning and preimplantation genetic diagnosis (3). On the other hand, if a gene is expressed in a particular stage of spermatogenesis, it will be possible to predict the progression of spermatogenesis through molecular methods and adapt it to histopathological findings. Therefore, study of these transcripts is important in the molecular identification of the spermatogenesis stage, oocyte fertilization and early stages of fetal development, as well as the association of genes with male infertility phenotype and its application in diagnostic procedures.

### Literature search

This review study was conducted on over 95 articles published in the Google Scholar, PubMed, Scopus, IranMedex, MEDLIB, IranDoc and Scientific Information Database (SID) for the comprehensive information on the biomarkers introduced for male infertility. All articles were reviewed by the keywords of transcript, sperm, semen, testicular tissue and infertility, until September 2018 and among them, 74 related papers were included. 

### Spermatic transcripts

RNA evaluation in sperms is recommended because it may show a historical record of spermatogenesis. Additionally, it can be considered as genetic background as well as fingerprint of the individual. Therefore, some RNAs may be brought up as potential diagnostic tools for evaluating male infertility and they may also play an important role in the development of fetuses and zygotes. 

### Coding RNAs

Dynamic cellular diversity has been reported in the RNA profiles of fertile and infertile men, and therefore scientists refer to it as biomarker of infertility. Round spermatids contain numerous varieties of transcripts that are stored in the spermatid cytoplasm before expression of the related proteins. In the middle of spermatogenesis, chromatin remodeling results in genome transcriptional inactivation. Therefore, most RNA transcripts were transcribed before the inactivation process. Using techniques such as real-time PCR, presence of the transcripts in human spermatozoa was confirmed (4). Data evaluation, using microarray, showed that adult human sperm has about 5000 types of mRNA molecules, expression of which vary about 10% between different specimens (5). The semen mRNA content can provide valuable information about the condition of spermatogenesis in the patient's testis, which cannot be detected by the conventional histopathologic methods. Although their possible roles are not revealed, many hypotheses could be proposed to illustrate the presence of mRNAs in sperm. Most evidences suggest that transmission of these mRNAs to oocyte may be as important as transfer of the haploid genome. Moreover, it is suggested that some paternal traits are transmitted to the child through the contents of sperm transcripts. If these transcripts play a role in the early differentiation of the fetus, these findings could be useful in advancing the technology of somatic cell nuclear transfer in cloning and also identifying effective factors in the development of infertility. Recently, it has also been shown that the amounts of sperm mRNA are transferred to the egg during fertilization and where their related proteins are synthesized. Therefore, it seems that transcripts of sperm have vital role in fetal development of the early stages (6). Researchers analyzed the RNA profile of sperm and testis in normosperm patients. They suggested that RNA profile is valuable to be used, regarding that can be used as a genetic fingerprint in fertile and infertile individuals and reflect past events during spermatogenesis. Development of the new research methods such as microarray and RNAseq can be useful as additional diagnostic tools and prognosis, for fertility and pregnancy. So far, based on the role of genes in spermatogenesis, numerous gene expression analyses were carried out on different specimens to determine the associated genes (Table 1). In many studies, expression of specific testicular genes has been analyzed, some of which are described below.

**Table 1:** Gene expression analysis in sperm, testis tissue and semen of fertile and infertile men


Gene name	Function	Sample type		AssociationP value	Ref.

DNMT1, DNMT3A, and DNMT3B	Methylation of DNA	Semen		No	(7)
RXFP3	Peptide receptor	Spermatozoa		No	(8)
PLCζ	Phospholipase (tes‌tis-specific)	Semen		Yes	(9)
PLCζ	Phospholipase (tes‌tis-specific)	Sperm		YesP≤0.05	(10)
PLCζ and CAPZA3	Phospholipase (tes‌tis-specific)/F-actin capping protein	Semen		Yes	(11)
PLCζ and PAWP	Phospholipase (tes‌tis-specific)/Meiotic resumption	Semen		Yes	(12)
PLCζ, PAWP and TR-KIT	Phospholipase (tes‌tis-specific)/Meiotic resumption/KIT proto-oncogene receptor tyrosine kinase	Semen		Yes	(13)
PAWP	Meiotic resumption	Semen		YesP<0.05	(14)
TR-KIT	KIT proto-oncogene receptor tyrosine kinase	Semen		YesP<0.01	(15)
JMJD1A	Demethylase	Tes‌tis tissue	Yes		(16)
PRM1, PRM2, YBX2 and JHDM2A	Compact sperm DNA (tes‌tis-specific)/DNA- RNA-binding protein (tes‌tis-specific)/Demethylase	Tes‌tis tissue	Yes/No for JHDM2A		(17)
YBX2 and JHDM2A	DNA- RNA-binding protein (tes‌tis-specific)/Demethylase	Tes‌tis tissue	Yes/No for JHDM2A		(18)
YBX2	DNA-RNA-binding protein (tes‌tis-specific)	Tes‌tis tissue	Yes P<0.0001		(19)
PRM1, PRM2 and TNP2	Compact DNA sperm (tes‌tis-specific )/Replacement of his‌tones to protamine (tes‌tis-specific)	Semen	PRM1, PRM2decrease/TNP2 increase		(20)
PRM1 and PRM2	Compact DNA sperm (tes‌tis-specific)	Tes‌tis tissue	Yes, for PRM1 P<0.001		(21)
PRM2	Compact sperm DNA (tes‌tis-specific)	Semen	No		(22)
Casp 9 and PRM2	Apoptosis/Compact sperm DNA (tes‌tis-specific)	Semen	Yes, for PRM2 P<0.05		(23)
KDM3A and PRM1	Demethylase/Compact sperm DNA (tes‌tis-specific)	Tes‌tis tissue	Decrease in NOA		(24)
DAZ, AKAP4, PRM1 and PRM2	RNA-binding protein/Regulatory subunit of protein kinase A/Compact DNA sperm (tes‌tis-specific)	Semen	Yes, for DAZ and PRM2		(5)
PRM1 PRM2 and HILS1	Compact DNA sperm (tes‌tis-specific)/Linker his‌tone	Sperm	Yes, for PRM1 and PRM2 P<0.001		(25)
ZMYND15, TNP1, PRM1 and SPEM1	Transcriptional repressor/Replacement of his‌tones to protamine (tes‌tis-specific)/Compact sperm DNA (tes‌tis-specific)/Spermatid maturation (tes‌tis-specific)	Tes‌tis tissue	Yes		(26)
PRM2, HSP90 and WNT5A	Compact sperm DNA (tes‌tis-specific)/Chaperone/Signaling proteins	Sperm	Yes P≤0.05		(27)
TNP1	Replacement of his‌tones to protamine (tes‌tis-specific	Semen	Yes ---		(28)
HSPA2	Folding and transport	Semen	No		(29)
TGIFLX/Y	Transcription factor (tes‌tis-specific)	Tes‌tis tissue	Yes		(30)
SYCP3	Recombination	Tes‌tis tissue	Yes		(31)
Septin14	GTP-binding cytoskeletal proteins	Tes‌tis tissue	Yes		(32)
DAZ	RNA-binding protein	Tes‌tis tissue	Yes		(33)
TSGA10	Sperm tail fibrous sheath	Tes‌tis tissue	Yes		(34)
Clus‌terin	Chaperone	Tes‌tis tissue	Yes		(35)
hTSH2B	His‌tone	Tes‌tis tissue	Yes		(36)
BAX and BCL-2	Apoptotic regulators	Semen	No/Yes		(37)
ERα	Es‌trogen receptor	Sperm	Yes P≤0.05		(38)
		Semen	Yes		(39)
SREs	Sperm RNA elements	Sperm	P≤0.05		(40)


### Sperm associated oocyte-activating factors genes

In about 1-3% of cases, failure of fertilization is due to the absence of sperm associated oocyte-activating factors (SAOAFs) in the posterior acrosomal region of the sperm head. During normal fertilization, when the sperm enters the egg, the egg is activated. This is associated with an increase in the concentration of calcium in the cytoplasm. Studies showed that increased intracellular calcium concentrations of oocytes are due to spermatozoa SAOAFs, including the *phospholipase C ζ (*PLCζ*), postacrosomal sheath WW domain-binding protein (PAWP)* and *KIT proto-oncogene receptor tyrosine kinase* (*KIT-Tr* proteins), which initiate the cascade of oocyte activation signal. **PLCζ** gene in humans, located at 12p12.3, is a family of phospholipase C enzyme. *PLCζ* protein is a special sperm protein with catalytic and domains catalytic X, Y and the Y-X binding region. At present, researchers often identify *PLCζ* as the most likely candidate for SAOAFs (41).

In the research performed by Park et al. (42), it was revealed that low expression of *PLCζ* was related to the oxidation of DNA sperm in human. Heytens et al. (43) showed that expression of *PLCζ* in infertile cases is lower due to the reduced fertilization rates. It may be suggested that the cause of fertilization failure after ICSI, may be due to the decrease or absence of *PLCζ* protein in some infertile people; therefore, they introduced this protein as a biomarker for fertilization failure. By studying this biomarker in infertility centers, an artificial oocyte activation (AOA) treatment method can be used to increase chance of improving fertilization rates in these individuals. Javadian-Elyaderani (11) demonstrated that due to the presence of a mutation in the vicinity of *PLCζ*, expression level of this gene was significantly reduced in infertile men with history of failed oocyte activation compared to normal men.

In addition, findings of Aghajanpour et al. (9) showed that expression of *PLCζ* was significantly lower in globozoospermic men or individuals with previously low or failed fertilization, in comparison with the control group. On this basis, they suggested that assessment of relative *PLCζ* expression may provide a useful marker for the ability of sperm to induce oocyte activation after ICSI. Unlike *PLCζ*, the exact molecular mechanism of the *PAWP* signal pathway is yet unknown. *PAWP* position in mammals was identified in the posterior acrosomal sheath of the sperm head. *PAWP* has no enzymatic activity, but it has hydrolytic activity on *PLCζ*. It is proposed that *PAWP* affects oocyte by interaction with other proteins. The results of these experiments showed that sperm injection with anti-*PAWP* antibody resulted in fertilization inhibition. Therefore, role of *PAWP* was considered as an oocyte activator. Abadi et al. (12) investigation showed that expressions of both *PLCζ* and *PAWP* were significantly reduced at RNA and protein levels of oligozoospermic men. They concluded that one of the reasons of fertilization failure after ICSI is due to the high percentage of sperm with small acrosomes and reduction of SOAFs might be associated with genetic abnormalities, such as mutations and gene deletions related to globozoospermia. The results of Tavalaee and Nasr-Esfahani (13) experiments were similar to those of the previous review. It was showed that expression profiles of *PLCζ* and *PAWP* were low in globozoospermic individuals.

On the other hand, in the study of Ghazavi-Khorasgani et al. (44), relative expression of *PAWP* was compared between varicoceles and fertile individuals at both mRNA and protein levels. Results showed that levels of *PAWP* mRNA and protein were decreased significantly in varicocele compared to fertile men. Therefore, one of the infertility etiologies in men with varicocele can be related to the decreased *PAWP* levels and inactivation of oocytes due to the effect of the increased testicle temperature on the expression of genes during spermatogenesis.

### Compacting DNA sperm genes 

**Fig.1:**
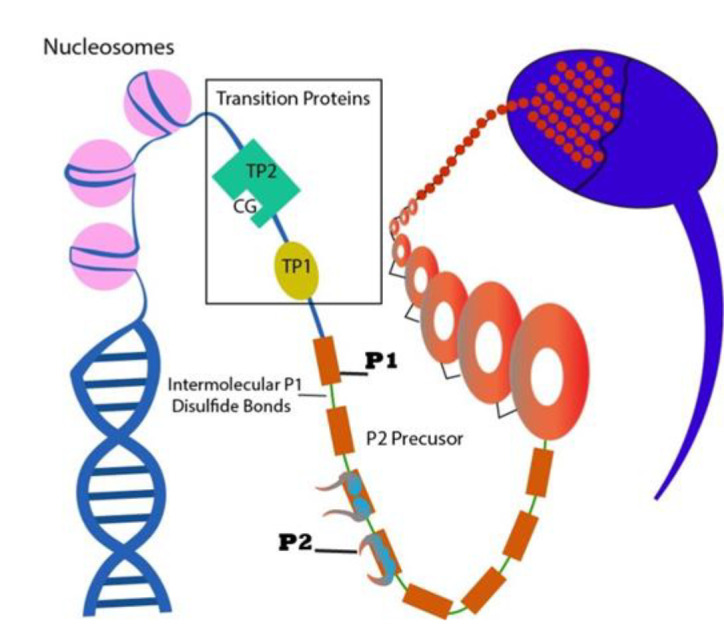
chematic representation of the sperm epigenetics. Hyper-acetylation of histones and activation of topoisomerase, to induce double-strand DNA breaks, allow histones to be replaced with transition proteins 1 and 2 (TP1 and TP2). Transition proteins are subsequently replaced with phosphorylated protamine, *PRM1 and PRM2*, which induce DNA compaction within the nucleus and form the nucleosome-bound chromatin. PRM1 is synthesized as a mature precursor, whereas the *PRM2* is generated by a partial processing of a single *PRM2* precursor (illustrated by the author).

Sperm transcripts play a dynamic role in reorganization of sperm chromatin. At the stage of spermatogenesis, somatic histones are replaced by transient proteins (TNP1 and TNP2) and then with protamine (*PRM1 *and *PRM2*) (Fig .1). Protamine is one of the most prominent and smallest sperm nucleolar proteins that are conserved amongst different species. In dense and mature spermatids, protamine proteins are a substitute for transient proteins and they are associated with genomic DNA (45). In Iranian research, it was found that *PRM1/PRM2* mRNAs ratio differed significantly among azoospermic men and normal group. Based on similar researches, it was proposed that decrease in the expression of *PRM2* gene could lead to male infertility. In line with the mentioned study, a survey showed (17) that *PRM2* down-regulation occurred much more than *PRM1 *in the sperm of infertile men. Although in Lambard et al. (46) study, increase of PRMT1 expression was reported in a low motile population. Due to the relation of protamin expressions with quality of sperm, they serve as biomarkers for diagnosis of male infertility. Results of several studies showed a significant relationship of sperm morphology with quantity of *PRM1, PRM2* and *TNP2* transcripts. Studies revealed significantly lower protamine transcript content in infertile fertile men (47).

Rogenhofer et al. (47) explained that *PRM1/PRM2* mRNA ratio in ejaculated spermatozoa could differentiate infertile from fertile groups. In terms of *TNP2*, Savadi-Shiraz et al. (20) reported a significant positive correlation between expression of *TNP2* gene and teratozoospermic samples, to compare with the control group (P<0.001) and sperm-head defects (P<0.05). Results of study performed by Liu et al. showed that normal development of sperm required microRNA-122 to control frequency of *TNP2* mRNA and its subsequent translation (48).

### Transcription factor genes (*TGIFL* and *YBX2*)

HOX genes, encoding transcription factors, play important roles in growth and development of mammals. Homeobox-containing genes (*TGIFLX/Y*) are members of this family and expressed in the testicles of mature males. However, their function is unknown and needs to be investigated (49). Aarabi et al. (30) evaluated the expression of *TGIFLY* in 110 azoospermic men and found no significant relationship between the gene expressions and spermatogenesis progression. One of the reasons for this finding is variation of the *TGIFLY* gene expression in different spermatogenesis stages, causing genetic heterogeneity in male infertility screening.

Y-Box proteins are DNA and RNA-coupled proteins that play role in controlling gene expression. According to the animal studies, expression of the *pmr1* and *tnp2* genes containing *Y-box* in the promoter was controlled by this mechanism and null mice showed a significant reduction in YBX2 expression (50). Moghbelinejad et al. (51) evaluated association of low levels of *PRM* mRNA and *YBX2* gene expression in testicular tissues of azoospermia men. They showed a significant correlation between reduction of *YBX2* gene expression and low level of *PRM2* deficiency in testicular spermatozoa in infertile men. Hammoud et al. (52) explained that the loss of *YBX2* had no effect on transcription, splicing or intracellular mRNA transport, but instead it had a selective effect on the translation rate. With regards to Iranian population, results of Najafipour et al. (18) showed a significant reduction of *YBX2* mRNA level in samples with impaired spermatogenesis (P<0.001) compared to control group.

### Non-coding RNAs

Duplication and unsuccessful differentiation of germ cells are the main causes of infertility and they are accomplished by regulating transcription of particular genes. Non-coding RNAs, such as microRNAs and long non-coding RNA (lncRNAs) are the main regulators of the expression of genes. The data obtained from deep-sequencing recently shows that lncRNAs are far more numerous than protein-coding RNAs, thus proving that the human genome is more active in terms of transcription compared to the previous view. Human testis tissue and immature sperm have 7% miRNAs and 17% piRNAs. These small RNAs regulate gene expression at the transcriptional, post-translational and chromatin levels. So far, it has been shown that one-third of human genes are regulated by miRNAs. In terms of numbers, more than 200 miRNAs have thus far been found in human sperm, which indicates the important role of these RNAs in morphogenesis and sperm maturation (53). Some non-coding RNAs associated with infertility in men are described below.

### microRNAs

micro-RNAs (miRNAs) have been introduced as the key regulators of gene expression at translation level and control of post-translation changes. Several studies showed that these miRNAs interfere with spermatogenesis in controlling pathways that affect human reproduction, such as the survival of primordial germ cells and spermatogenesis. miRNAs are existed in various stages of spermatogenesis and they have great expression in spermatid and spermatocyte pachytene cells. They exist in the body fluid in combination with lipoproteins or they are enclosed in packages of double-layer membranes called exosomes. Focus on the role of microRNAs in male reproductive disorders can further explain the molecular mechanisms of male infertility and it can create a new pathway as an effective biomarker for treating infertility in men and for contraceptive pills (54). So far, extensive studies have been conducted (55) to determine level of different miRNA expressions and association of their polymorphisms with male infertility (Table 2).

For instance, by examining two miRNAs (miR-100 and let-7b) regulating the alpha estrogen receptor gene in oligospermia men, Abhari et al. (56) found that the expression level of both miRNAs were significantly increased (P=0.008 and P=0.009 respectively) leading to decrease in the expression of alpha estrogen, while estrogen plays a key role in spermatogenesis. They also found a significant expression change in miR-99, miR-196, miR-21 and miR-22 (39). 

**Table 2:** Non-coding miRNAs expression in infertile men


Name	Sample size(case-control)	Sample type	Association	P value	Ref.

miR-21	43-43	Semen	Yes	P<0.0001	(39)
miR-22	43-43	Semen	Yes	P<0.0001	(39)
miR-100	43-43	Semen	Yes	P=0.008	(56)
let-7b	43-43	Semen	Yes	P=0.009	(56)
miR-34c	55 totally	Semen	Yes	----	(58)


**Fig.2:**
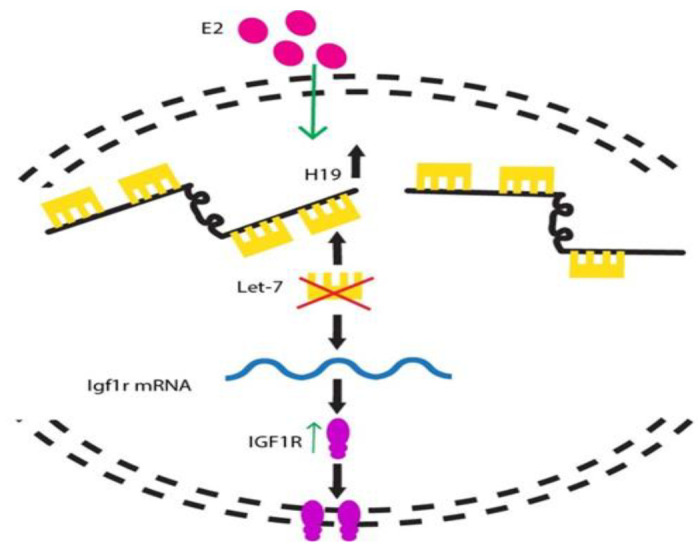
*H19* is a long noncoding RNA (lncRNA) that plays role in cell growth via the microRNA let‐7. Decreased *H19* expression leads to increased activity of let‐7. Aberrations in the *H19*/let‐7 regulatory pathway may represent one potential mechanism for male infertility. On the other hands, in the paternal allele of fertile men, *H19* leads to IGF2 expression. Repression of *H19* transcription increase IGF1R expression. Both IGF2 and IGF1R transcripts are involved in sperm capacitation and embryo growth (illustrated by the author).

Current investigations disclosed that let-7b has an inhibitory effect on cell proliferation (Fig .2) (56). The relationship of ER expressions with miR-7b, miR-21 and miR-22 were previously reported in other diseases (57). In another study, Rahbar et al. (58) showed statistically significant increased expression of miR-34c in moderate oligoasthenoteratozoospermic and non-obstructive azoospermia.

Bouhallier et al. (59) demonstrated that miR-34c was highly expressed in mouse germ cells and therefore, they introduced it as a promising candidate gene for test in male germ cell cancer and sterility.

### piRNA

**Fig.3:**
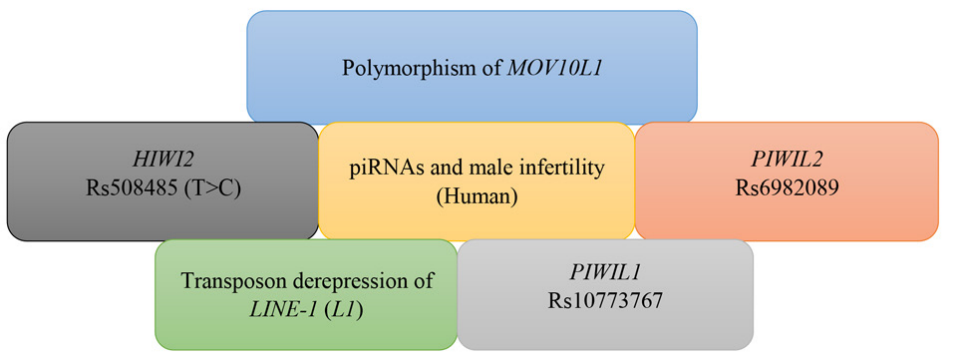
The genes associated with polymorphisms of piRNAs and male infertility. The latest researches on the relationship between piRNAs and human male infertility are showed in this figure (illustrated by the author).

piRNA is a group of ncRNAs that act through their interaction with Piwi protein. It has 24-30 nucleotides and specific expression in the testicular tissue as well as the sex cells, while it has not been identified in mature sperm. This group of ncRNAs is often found among clusters of repetitive sequences in the genome and it is not translated into proteins (60). Some of these molecules include PRG-1, HIWI, MIWI2 and piRNA. PRG-2s are produced at the pachytene stage of spermatocytes. There are several reports suggesting that piRNAs protect germ cells from the retrotransposons. Similar to miRNA, piRNAs are the molecules that play important role in the regulation of the post-translation process of germ cells. They are expressed in spermatocytes. They play important role in inhibiting retrotransposition and regulation of gene expression after transcription during meiosis. It has also been shown that due to the role of piRNAs and miRNAs in the pathway of spermatogenesis, applying their inhibitors leads to disturbances in spermatogenesis, in addition to prevention of pregnancy (61). According to the literature, few investigations has been performed in Iran to determine role of piRNAs. In 2010 and 2017, researches on the Iranian and Chinese population disclosed cases and it can be proposed as a risk factor of male infertility (62). The association between *HIWI2* rs508485 (T>C) with non-obstructive azoospermia and *HIWI2* rs508485 (T>C) with non-obstructive azoospermia (male infertility in china) was previously confirmed in the Iranian and Chinese populations. Figure 3 summarized the most prominent study carried out on piRNAs and male infertility: moloney leukemia virus 10-like 1 (*MOV10L1* is a gene involved in piRNA biogenesis, playing a key role in primary and secondary function (63). *MOV10L1* may participate in the binding of primary piRNAs to the PIWI proteins. Several researches approved that many polymorphisms of *MOV10L1* caused a significant enhancement in men’s infertility (64).

### lncRNAs

lncRNAs are dark matter of the genome. So far, a small number of lncRNAs have been studied in male infertility and function and expression pattern of many are yet unknown. Using next generation sequencing (NGS) technology, one of which is RNAseq, shed light on the molecular mechanism involved in infertility disorders, especially structural and functional defects in sexual cells. It is also believed that lncRNAs affect the proteins involved in sperm motility and its entry into the egg as well as the pathways and mechanisms involved in fertility. Therefore, by studying and recognizing these relationships as well as the manipulating and molecular interventions, new approaches and diagnostic-therapeutic kits can be designed. In the study performed by Zhang et al. (65), expression of *HOTAIR* in asthenozoospermic and oligoasthenozoospermic patients was significantly decreased in comparison with the control group. They also found that expression of *HOTAIR* was associated with sperm function parameters, including motility and vitality. On the other hand, Rinn et al. (66), demonstrated that lncRNA *HOTAIR* interacted with the polycomb complex PRC2, which methylates histone H3K27 to promote gene repression.

## Discussion

Proper and complete spermatogenesis requires simultaneous expression of a very large number of coding and non-coding genes. So that stopping or disrupting expression of each one can lead to disruption of the spermatogenesis process. Identifying such genes and evaluating their performance provides valuable information about the role of these genes in adult sperm, process of spermatogenesis, their function in embryo fertilization and causes of idiopathic infertility. Genetic and epigenetic factors are elements contributed to this type of infertility.

Therefore, this aspect needs to be considered for the investigation of infertile men. The main emphasis for molecular evaluation and use of RNA sperm is the wide range contribution of male factor in infertility and testicular biopsy, which is a problem in the study of infertile men (67). Those who fail first sperm retrieval may be candidates for the second TESE, hoping to have their biological children. According to the success rate of 60% for TESE, a simple RNA analysis can help predict the relative success of sperm retrieval in biopsy and help in counseling and managing these cases. It may also help the surgeon predict the amount of necessary tissue for sperm retrieval for ICSI or diagnosis in future biopsies (68). Today, with remarkable advances in the field of molecular medicine, study of the RNA content in spermatids is possible by using techniques, such as RNAseq analysis and microarray, which reveals the combination of mRNA in adult sperm and relationship of the specific pattern of these transcripts with fertility and infertility in men. Clinical application of semen and germ cell RNAs is noticeable, due to the fact that sperm can provide the same information. Additionally, non-invasive sampling of the semen is better and more acceptable choice for the patient rather than biopsy (26). In a study performed to evaluate expression of the specific genes, including *AKAP, PRM2* and *DAZ*, it was showed that presence of *DAZ* and *PRM2* genes can be used as a noninvasive molecular marker in seminal fluid of non-obstructive azoospermia patients to predict the presence or absence of sperm or mature spermatids (5). Recent studies showed that miRNAs and their transcripts in the seminal fluid were used to investigate spermatogenesis in infertile men. Although mature sperm is silent in transcription, it contains a set of transcripts of non-coding RNAs and mRNAs that play special role in the early stages of embryonic development as an epigenetic effect. New results from microarray, NGS and RNAseq techniques led to the discovery of new transcripts in sperm and clinical markers of male infertility. It seems that gene expression profile in sperm can help identify the required sperm factors in early embryonic growth. Although the amount of sperm RNA is negligible, it is important for the investigation and diagnosis of male infertility. Transcripts that are specifically expressed in germinal cells and present in adult sperm are suitable molecular markers for the diagnosis of cell lines in spermatogenesis and they can provide a generalized picture of spermatogenesis in the infertile testis instead of invasive testicular biopsy. Evaluation and quantification of more transcript content of normal human sperm could provide the essential biomarkers for assessment of male fertility in the future, while new qualifications and methods must provide for changes in diagnosis (40).

## Conclusion

In the scope of infertility investigations, there are several available tests evaluating sperm quality and function. But, there is still a demand for better and more reliable procedures, considering that male factor infertility is involved in at least 45-50% of idiopathic cases. Use of sperm transcripts in molecular analysis of spermatogenesis and infertility treatment is also important, especially in patients with non-obstructive azoospermia. Studies showed that in these patients expression of genes in the pathway for sperm production is changed.

Today, the most common method for evaluating spermatogenesis in these individuals is testicular biopsy which is an invasive practice and recommended as an infertility study tool in the final stage. However, sperm may not be found due to regional spermatogenesis in the testicular tissue taken from the biopsy and need for multiple biopsies of the patient to find and extract the sperm. This procedure can cause tissue atrophy or infection. Thus, using sperm RNA content and preterm sexual cells can evaluate spermatogenesis molecular events. Clinical application of sperm RNA is very valuable because if sperm RNA can provide similar information compare to testis tissue, semen samples are a better and more acceptable choice for the patient than a biopsy. The existence of DAZ transcripts in the seminal fluid of non-obstructive azoospermia can be used as a noninvasive molecular marker to predict presence or absence of adult spermatozoa. From other futuristic studies, transformation of embryonic stem cells or adult stem cells into germinal cells can be a very valuable starting point for solving the infertility problem in an individual whose defect is related to the absence of stem cells in the testes. Research on the presence of RNAs in differentiated sperm from stem cells and normal sperm and their role in *in vitro* spermatogenesis can be suggested in the future.
